# Closing Editorial for the Special Issue: Cardiac Electrophysiology and Catheter Ablation of Different Arrhythmias

**DOI:** 10.3390/jcm14145055

**Published:** 2025-07-17

**Authors:** Hussam Ali, Antonio Frontera

**Affiliations:** 1 Arrhythmia & Electrophysiology Centre, IRCCS MultiMedica, Sesto San Giovanni, 20099 Milan, Italy; 2 Cardiac Arrhythmia Department, Great Metropolitan Hospital Niguarda, 20162 Milan, Italy

Cardiac electrophysiology and catheter ablation for diverse arrhythmic substrates continue to evolve rapidly, leveraging new techniques and energy sources. While significant advances in catheter ablation have enhanced safety and efficacy, many challenging arrhythmias and conditions still require further study and research in order to optimize patient outcomes.

As Guest Editors of the Special Issue “Cardiac Electrophysiology and Catheter Ablation of Different Arrhythmias”, we focused on the management, diagnosis, intracardiac mapping, and ablation of cardiac arrhythmias. This Editorial highlights key findings from this Special Issue and outlines future research directions.

Although the implantable cardioverter–defibrillator (ICD) is an established strategy for preventing sudden cardiac death (SCD) in heart failure (HF) patients, it can lead to significant complications, comorbidity, and reduced quality of life [1]. In this light, research on the optimization of HF medical treatment, with the aim of improving hemodynamics and cardiac remodeling and reducing the burden of ventricular arrhythmias, is critical. In their comprehensive and highly relevant review, Zaher et al. summarize the literature data concerning the antiarrhythmic effects of optimized medical therapy and their impact on SCD prevention in HF patients with reduced ejection fraction [2]. The authors confirm that there is robust evidence that a protective role is played by beta-blockers, mineralocorticoid receptor antagonists, and Angiotensin receptor–Neprilysin inhibitors and show promising data regarding the use of Na-glucose cotransporter 2 inhibitors. Notably, while current guidelines for ICD implantation rely heavily on remote trials, future research must evaluate how contemporary medical therapies influence SCD risk. This may refine ICD indications for primary prevention in HF patients [3].

Similarly to HF patients, individuals with hypertrophic cardiomyopathy (HCM) are also at an increased risk of SCD, but there are fewer evidence-based data for risk stratification and low agreement between the available guidelines for primary prevention [4]. In their comprehensive review on the topic, Sclafani et al. analyzed the complex mechanisms and substrates of SCD in HCM patients. Unlike traditional reviews, the authors propose a dynamic, physiology-driven framework for SCD risk prediction that incorporates cellular, structural, and electrophysiological substrates [5]. Cellular abnormalities, such as ionic dysfunction, may occur early in the disease, preceding overt structural abnormalities, which might explain poor outcomes in some HCM patients who received an “apparently” favorable prognosis based on current criteria. Conversely, while myocardial scarring is a recognized risk factor, its architecture/myocardial disarray and associated functional substrates are complex and remain challenging to assess. The authors emphasize that future advancements in genetic, molecular, and cardiac imaging diagnostic tools may provide a more individualized substrate-driven assessment of SCD risk and ICD indications in HCM patients and may guide specific non-invasive treatments.

Ventricular arrhythmias are a major cause of SCD, particularly in the context of underlying electrical or structural heart disease. While catheter ablation is often considered a curative therapy for idiopathic ventricular tachycardia, its role is usually adjunctive to ICD therapy in the presence of structural heart disease [6]. In this Special Issue, Schlatzer et al. present their “real-world” experience regarding catheter ablation of ventricular arrhythmias in 120 patients at a Swiss tertiary care center [7]. Their data confirm high acute success rates (>90%) for catheter ablation (CA) for both premature ventricular beats and ventricular tachycardias. On the other hand, related complications (<10%) were mainly observed in patients with structural heart disease. In this light, the preventive role of CA in patients with structural heart disease and its potential substitution for ICD therapy require further assessment in large randomized clinical trials.

Another patient cohort with an increased SCD risk comprises those with ventricular pre-excitation (Wolff–Parkinson–White, WPW), mainly due to the rapid conduction over their accessory pathway during atrial arrhythmias. Though CA has been proven to be a safe and curative therapy in most WPW patients, its timing and related complications should be carefully assessed in children [8]. In this regard, Sanzo et al. present important findings from their retrospective study including 153 pediatric WPW patients [9]. At a long-term follow-up of about 5 years, the spontaneous loss of pre-excitation was observed in 28% of patients. Interestingly, left-truncated Kaplan–Meier analysis estimated that pre-excitation would persist only in about one half and one third of patients at the age of 1 and 16 years, respectively. Predictors of the spontaneous resolution of pre-excitation included an intermittent WPW pattern and the absence of symptoms. Notably, no malignant arrhythmic events were reported in the study cohort. Accordingly, these findings support a more conservative approach in children with WPW since spontaneous pre-excitation loss occurs frequently in the early years, obivating the need for invasive treatments.

Atrial fibrillation (AF) is the most common sustained arrhythmia, with a substantial impact on morbidity, mortality, stroke risk, quality of life, and health system capacity. Despite impressive advancements in CA as a promising therapy, numerous challenges are still present, with suboptimal ablation outcomes observed in many AF patients [10].

While an in-depth and comprehensive understanding of AF mechanisms, particularly persistent forms, is still lacking, most of the current research and trials are orientated towards ablation “burning” rather than mapping “understanding”. In their review paper, Ye et al. navigate the fascinating field of AF mapping using high-density epicardial mapping [11]. The authors highlighted the importance of accurate unipolar atriogram analysis, mapping during both sinus rhythm and AF, and the role of Bachmann’s bundle and endo-epi asynchrony in AF maintenance. Though epicardial mapping and signal fingerprinting may be limited to cardiac surgery and research studies, combining them with rapidly evolving artificial intelligence may improve our understanding of AF mechanisms and substrates, offering novel ablation targets for a tailored approach [12]. We congratulate the authors’ group, guided by Prof. De Groot, on their continuous research focusing on signal analysis and decoding AF mechanisms.

An additional innovative AF mapping technology is electrocardiographic flow (ECF) mapping, which allows a temporospatial display of complex atrial propagation patterns during AF, helping to localize active drivers or “hot spots” [13]. In their analysis of two previous studies, Nilsson et al. show the utility of this approach in phenotyping the AF substrate and predicting recurrences over a 12-month follow-up after AF ablation. Patients with untreated non-pulmonary vein sources, identified through ECF, had higher rates of AF recurrence [14]. Future research is needed to assess the utility and feasibility of ECF mapping in daily practice, particularly in guiding individualized ablation in persistent AF substrates.

Atypical (non-cavotricuspid isthmus-dependent) atrial flutters may present a proarrhythmic effect from previous AF ablation. Although less complex than AF substrates, atypical flutters can be challenging and often require advanced skills and high-density mapping [15]. In a retrospective study including 19 patients, Johner et al. elegantly displayed the electrophysiological features and substrates of 25 atypical atrial flutters using a 3D mapping system (Rhythmia) [16]. More than 50% of circuits were dual-loop re-entry circuits, mostly involving a perimitral loop with a second roof-dependent loop. Interestingly, dual-loop circuits showed wider isthmuses and faster conduction velocities. Ablation at the common isthmus of a dual-loop re-entry more frequently terminated the arrhythmia than targeting uncommon isthmuses, which often led to a change in tachycardia rate or activation sequence. This timely article highlights the importance of high-density mapping combined with conventional pacing maneuvers as multisite atrial entrainment in accurately delineating the entire tachycardia circuit and identifying the optimal ablation target.

Due to the progressive nature of AF (i.e., AF begets AF), the timing of CA could play a critical role. In their retrospective study, Farghaly et al. assessed the impact of CA timing in 130 AF patients undergoing RF pulmonary vein isolation, with a 16-month median follow-up [17]. The early-ablation group (within one year of AF diagnosis) had higher rates of freedom from atrial arrhythmia recurrence (84.4% vs. 60.8% in the late-ablation group, *p* = 0.039). Moreover, early ablation was associated with lower rates of cardiovascular hospitalizations (18% vs. 42%, *p* = 0.023) and AF progression (0.0% vs. 11.3%). According to multivariate analysis, late ablation and AF recurrence during the blanking period were independent predictors of recurrences. These findings were confirmed in a recent meta-analysis including more than 40.000 AF patients over 2 years of follow-up [18]. Early ablation was associated with lower recurrence rates in both paroxysmal and persistent AF, particularly in younger patients (<55 years) [18].

One of the challenging aspects of CA in AF is the creation of transmural durable lesions, with the aim of permanently isolating the pulmonary veins without compromising procedure safety. Over the last several years, advancements in CA technologies and energies, 3D mapping systems, and operator experience have contributed toward addressing this challenge [19]. Interestingly, Sprenger et al. used a complex machine learning model to predict ablation or lesion size indexes for local-impedance drop-guided ablation, showing significant differences in application duration through the different metrics used [20]. Although this study was retrospective and requires affirmation through future studies, it highlighted the promising utility of machine-learning in predicting ablation lesion quality using multi-parametric data.

Pulsed-field ablation (PFA) is an emerging ablation technology that has rapidly attracted attention in clinical practice due to its remarkable efficacy and safety profile. Beyond its fast and efficient workflow during AF ablation, the key advantage of PFA lies in its cardiac selectivity, which helps to minimize collateral damage and enhance procedural safety [21]. In this Special Issue, Mahrous et al. assessed left atrial function following PFA of AF in a limited number of patients over a six-month follow-up [22]. Their findings demonstrated improved echocardiographic parameters of left atrial function after ablation, indicating favorable atrial remodeling. Notably, these preliminary data have been corroborated in patients with persistent AF undergoing more extensive PFA beyond pulmonary vein isolation (such as posterior wall isolation) without any negative impact on left atrial compliance and function [23].

In summary, this Special Issue discusses several pressing topics in the rapidly evolving field of cardiac electrophysiology and catheter ablation, including ventricular arrhythmias, sudden cardiac death, and atrial fibrillation. We extend our sincere thanks to all the authors, reviewers, and *Journal of Clinical Medicine* staff for their valuable contributions and support in bringing this Special Issue to fruition.
